# Concomitant bilateral lung transplantation and diaphragmatic hernia repair: A case report

**DOI:** 10.1016/j.xjtc.2026.102324

**Published:** 2026-03-20

**Authors:** Patrick McGeoghegan, Gabriel Loor, Erik Eddie Suarez, Nirmal Sharma, Gloria Li, Jorge Rodriguez, Aladdein Mattar, Abdussulam Elsenousi, Ramiro Fernandez

**Affiliations:** a Division of Cardiothoracic Transplantation, Baylor College of Medicine, Houston, Tex; b Division of Pulmonary Medicine, Baylor College of Medicine, Houston, Tex; c Pasadena Surgical Association, Pasadena, Tex

**Figure:**
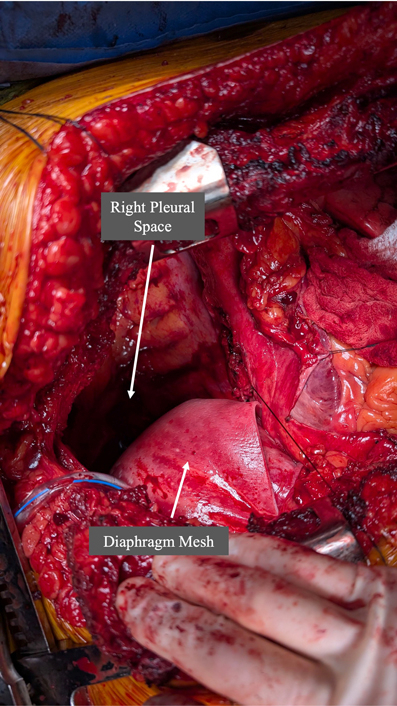
Right pleural space after diaphragmatic hernia repair and pneumonectomy.

**Central Message**

Diaphragm repair is feasible at the time of lung transplantation.

Patients with end-stage lung disease requiring bilateral lung transplantation (BLT) may have coexisting pathologies that complicate the operative approach. We describe a surgical strategy that we used to effectively manage a patient requiring BLT and a large diaphragmatic hernia. The patient provided written informed consent for publication. Institutional review board approval was not required.

## Case Presentation

**Figure 1:**
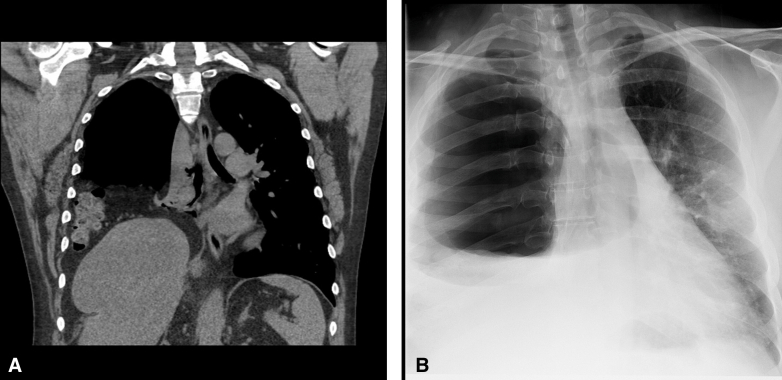
A, Computed tomography of the chest showing a large right-sided diaphragmatic hernia. B, Radiograph of the chest demonstrating right-sided bulla with mediastinal shift.

A 55-year-old man with end-stage emphysema was evaluated for lung transplantation. He had a history of traumatic right diaphragmatic hernia from a motor vehicle accident at age 19 years of age. Computed tomography scan showed centrilobular emphysema with a giant bulla filling the entire right pleural space producing mediastinal shift. There was a large central defect in the right diaphragm measuring 13 cm × 14 cm with herniation of the liver, transverse colon, and omentum (Figure 1, Video 1). He was listed for concomitant BLT and right diaphragmatic hernia repair.

**Figure 2:**
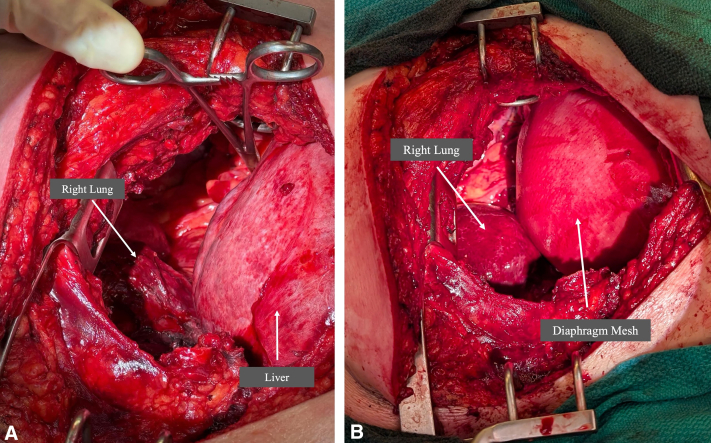
A, Large right diaphragmatic defect with exposed liver. B, Completed diaphragm repair.

The diaphragm repair was performed first, followed by BLT. The diaphragm repair was approached via a right posterolateral thoracotomy in the seventh interspace. The posterior, lateral, and anterior rim of diaphragm were dissected off the liver. The omentum was resected with LigaSure (Medtronic) and the colon was reduced through an anteromedial defect (Figure 2). To repair the defect, a 20 × 30-cm STRATTICE (AbbVie) mesh was secured to diaphragm rim circumferentially with interrupted No. 1 ETHIBOND sutures (Ethicon; Figure 2, *B*). Because there was no diaphragm rim medially, the mesh was bridged over the mediastinum. Total operating time for the diaphragm repair was 4 hours.

Off-pump BLT was then performed via clamshell approach. LUNGguard (Paragonix) was used for donor lung preservation. Total ischemia time was 407 minutes for the right lung and 487 minutes for the left. The postoperative course was prolonged by deconditioning and high chest tube output but was otherwise uncomplicated, and the patient was discharged on postoperative day 24. The patient returned to work 3 months after transplantation and at 12-month follow-up demonstrated excellent graft function with a forced expiratory volume in 1 second of 2.85 L (64% predicted). One-year postoperative computed tomography demonstrated durability of the hernia repair (Video 2).

## Discussion

This case demonstrates the complex decision-making required to manage a technical obstacle in lung transplantation. Options included staged diaphragm repair, left single-lung transplant to avoid the right pleural space altogether, or concomitant diaphragm repair and BLT.

A staged diaphragm-first strategy risked rupture of the giant bulla with subsequent bronchopleural fistula physiology that would have been difficult to manage. Conversely, staged BLT before hernia repair risked graft compression by visceral contents. Given the patient's age, BLT rather than single-lung transplant offered superior long-term survival.^1^ Consequently, a concomitant diaphragm repair and BLT was selected as the ideal management strategy.

We considered diaphragm repair through standard lung transplant approaches such as clamshell or bilateral thoracotomies in the fourth or fifth interspace.^2^ However, because the liver had herniated into the chest and was in a fixed position, the remaining 2 cm rim of diaphragm was very deep and difficult to expose for safe mobilization off the liver from a fourth interspace clamshell or fifth interspace posterolateral approach. Therefore, we elected for a separate low posterolateral thoracotomy in the seventh interspace, which provided excellent exposure for diaphragm repair. A mesh repair was necessary to prevent re-herniation of visceral contents. We considered both absorbable and permanent mesh options. Although both types of mesh pose a risk for early infection, we elected to use a bioabsorbable mesh to reduce the risk of late infection. Recipients of lung transplants remain on extended antibiotic prophylaxis; therefore, no changes to the standard regimen were made.

Controlled hypothermic preservation with the LUNGguard device allows for safe extension of cold preservation time and provided an ischemia time safety margin to accommodate the unpredictable total operating time without compromising graft quality.^3^ This proved useful because the diaphragm repair added 4 hours to the total operating room time. Allograft function was excellent, and the patient recovered with significant improvements in quality of life. In summary, competing thoracic pathologies may necessitate creative operative planning to offer patients lifesaving lung transplantation.

## Conflict of Interest Statement

G.L. is co-founder and medical officer for OrganVive. Baylor College of Medicine receives institutional grant support from Transmedics. All other authors reported no conflicts of interest.

The *Journal* policy requires editors and reviewers to disclose conflicts of interest and to decline handling or reviewing manuscripts for which they may have a conflict of interest. The editors and reviewers of this article have no conflicts of interest.

## Supplementary Data

Video 1 CT of the chest demonstrating the large bulla filling the right pleural cavity and the right diaphragm hernia involving liver, colon, and omentum. Video available at: https://www.jtcvs.org/article/S2666-2507(26)00131-8/fulltext.

Video 2 CT of the chest at 1 year demonstrating intact diaphragm repair. Video available at: https://www.jtcvs.org/article/S2666-2507(26)00131-8/fulltext.
