# Supplementary figures and images for: Concomitant bilateral lung transplantation and diaphragmatic hernia repair: A case report
Source: JTCVS Tech. 2026 Mar 20;37:102324. doi: 10.1016/j.xjtc.2026.102324 (PMC13261221; doi:10.1016/j.xjtc.2026.102324)

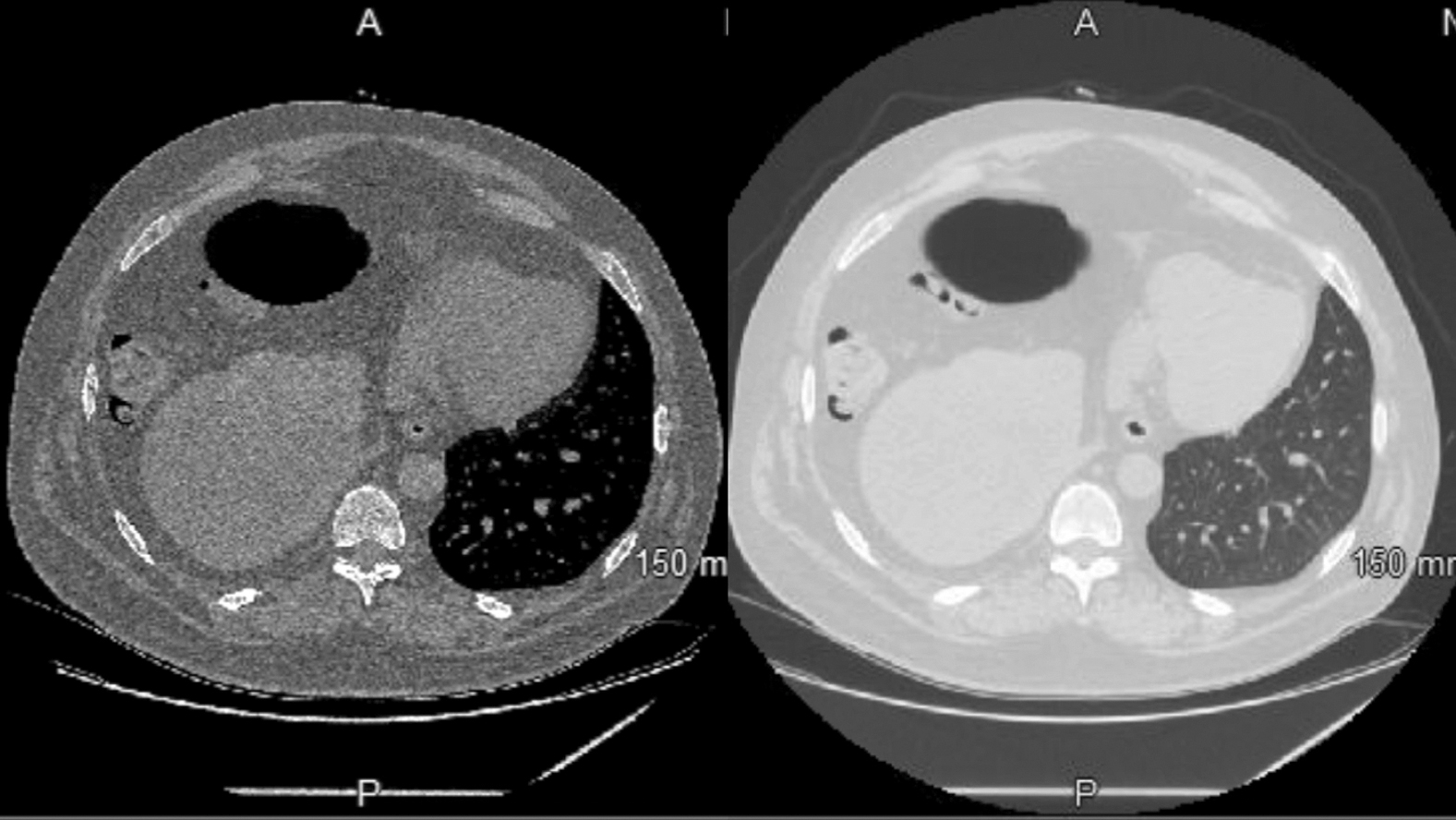

Supplement: Video 1 — CT of the chest demonstrating the large bulla filling the right pleural cavity and the right diaphragm hernia involving liver, colon, and omentum. Video available at: https://www.jtcvs.org/article/S2666-2507(26)00131-8/fulltext. [file fx2.jpg]

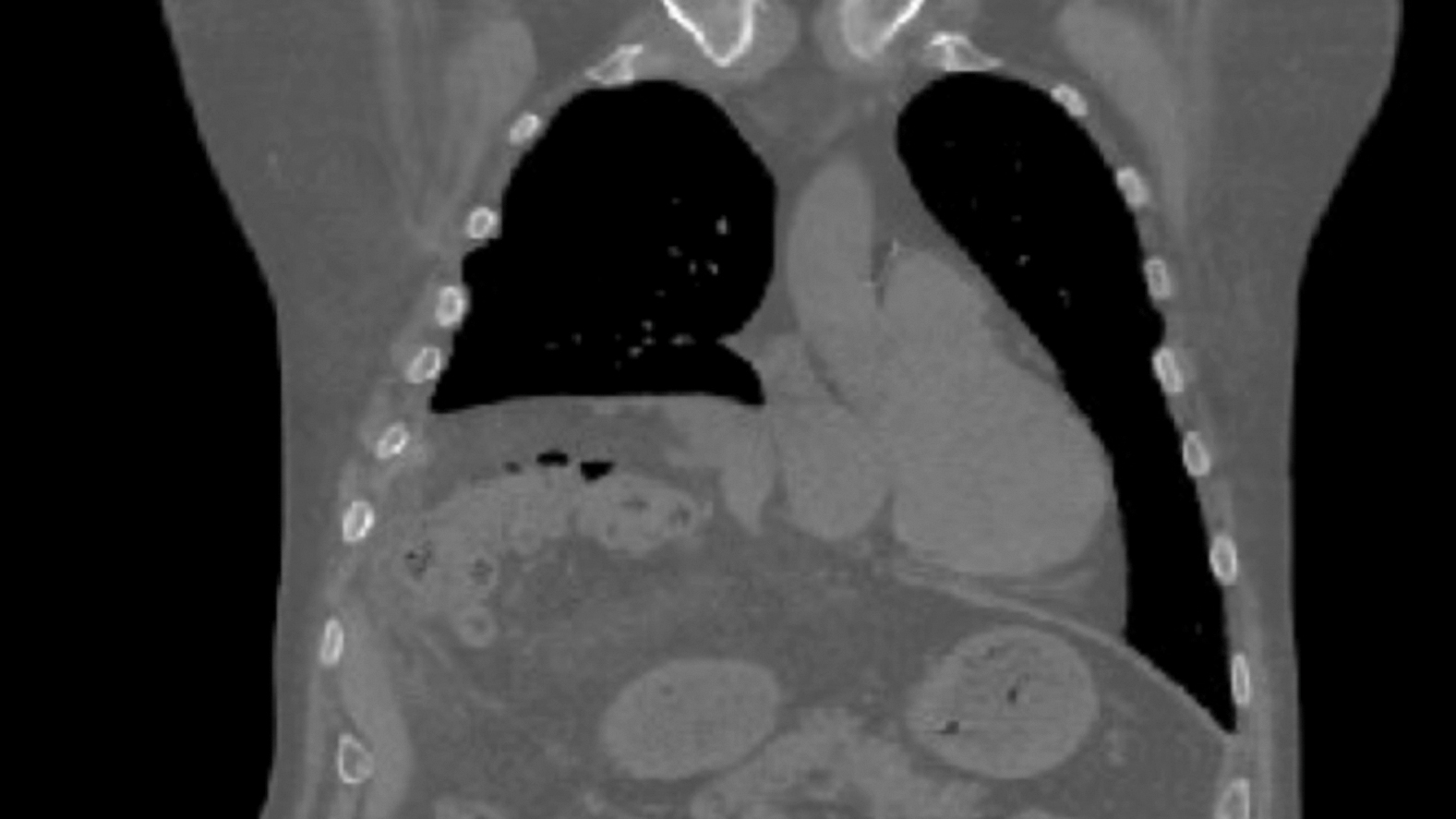

Supplement: Video 2 — CT of the chest at 1 year demonstrating intact diaphragm repair. Video available at: https://www.jtcvs.org/article/S2666-2507(26)00131-8/fulltext. [file fx3.jpg]
